# Learning style among family medicine residents, Qatar

**DOI:** 10.11604/pamj.2021.38.167.27668

**Published:** 2021-02-15

**Authors:** Amal Abdulla Al Ali, Mohamed Salem Nasrallah, Mostafa Hamdy Rashed, Yosaf Abdo Ibrahim, Rafea Muftah Rasheed, Hassan Mahmoud El-Meedani, Mohamed Soliman Abdel-Hamid, Hisham Al-Mahdi Mustafa

**Affiliations:** 1 Family Medicine Department, Primary Health Care Corporation, Doha, Qatar

**Keywords:** Learning style, family medicine, residents, Qatar

## Abstract

Different learning style among family medicine residents is important to adjust the educational program that meet their needs and make the educational process fruitful to improve their academic performance. This study is aiming to assess learning styles among family medicine residents in Qatar. This cross-sectional descriptive study was conducted at the West Bay family medicine training center, Doha, Qatar, where all family medicine residents were invited to participate using self-administered validated questionnaire based on David Kolb model of experiential learning that has been extensively used in medical education research. Demographic data were assessed and analyzed as the predictor variables. Data were collected from 38 residents with response rate 76% revealing that the predominant pattern in postgraduate year one (PGY1) is activist in 65% and theorist in 55% while PGY2 tends to be reflector in 45% and theorist in 35% and in PGY3-4 changed to be 70-75% activist and 40-55% (reflector and pragmatic). General learning style pattern among all residents tend to be in the following order: activist 60.5%, then reflector 44.7%, followed by pragmatism 34.2% and finally theorist 36.8%. Learning style assessment is important and can be used to determine which teaching modalities will be best accepted and most effective for family medicine residents which should be considered while planning, designing, and implementing their educational program.

## Introduction

With rapid increase of mass of information those days. Learning, an effective method to maximize the resident learning outcome can improve the current health status. Most of family medicine programs are focus on lectures-based teaching style while there are many different other methods to deliver knowledge to their post graduate residents. Different learning style is an important tool help the junior doctor to ease their residency journey. Very important is that family medicine program in Qatar understand the preferred methods of the learning styles among the resident in turn that facilitate instructional rapport between postgraduates and the physicians’ faculties which lead to improve the academic performances. Most resident complaints about the learning style which are applied by the department without involving them as the major decision maker.

Learning style can be defined as different and unique ways used by individuals as they prepare to learn and recall information [1]. The importance of knowing of postgraduate learning style in family medicine residency program is that, it can help to deliver the information in effective ways. There are many different learning style methods that the post-graduate finds as the most effective way of learning. Learning from health centers clinics, academic day lectures or even the rotation in headmasters' and headmistresses' conference (HMC). The main end results from knowing the preferred methods of learning methods is to get better care for patients who attending different health centers in Qatar. Learning styles are different and a lot of studies have been done to evaluate and assess the best style for educating the residents, for example there were two randomized controlled trial (RCT) done to compare between instructional and cognitive style in web base learning and the results showed: cognitive and learning styles had no apparent influence on learning outcomes. There were no differences in outcome between these instructional methods [2]. Furthermore, there was strong evidence to support the importance of combining the techniques of teaching as much attention has been given to learning and teaching “styles” in adult education and in medical education. Since style refers to assumptions and beliefs about how adults learn, it affects what teachers do to facilitate learning. A synthesis of two models view the educational process as a cycle: “experience,” followed by “reflection,” “abstraction,” and “experimentation.” Four styles of learning or teaching are possible by combining experience with observation, observation with abstraction, abstraction with experimentation, and experimentation with experience. Students and residents could use this information to negotiate with faculty to best meet their educational needs, and faculty could use this information to tailor their teaching strategies to best suit each encounter [3].

A cross-sectional study of internal medicine residents and faculty members at Morehouse School of Medicine was performed using the Kolb Learning Style Inventory (LSI) version 3.1 to reach the preferred learning style among residents and faculty members of an internal medicine residency program and the conclusion was the understanding of residents' learning styles may facilitate instructional rapport between residents and attending physicians, thereby improving residents' academic performance [4]. A retrospective descriptive analysis of academic learning logs submitted by residents as part of their academic training requirements done in London, onto, and showed that residents used a variety of learning modalities and chose self-study over other more traditional modalities (e.g., lectures) for most of their academic learning. A successful academic program must take into account residents' various learning preferences and habits while providing guidance and training in the use of more effective learning methods and resources to maximize educational outcomes [5]. Very important is that family medicine program in Qatar understand the preferred methods of the learning styles among the resident in turn that facilitate instructional rapport between postgraduates and the physicians’ faculties which lead to improve the academic performances.

## Methods

**Study design:** descriptive cross-sectional study.

**Study setting:** this study was conducted in West Bay Training Health Center affiliated to Primary Health Care Corporation in Qatar where the Family Medicine Residency Program runs its activity in the form of academic days and continuity care clinic which is run by residents of family medicine and supervised by faculties from the family medicine department.

**Study subjects:** it included all family medicine residents who accepted participation reaching 38 residents from 50 residents representing 78% response rate conducted during 2018.

**Data collection methods:** self-administered questionnaire including basic demographic data and the learning style questionnaire, it is 80 item questions. This questionnaire is designed to find out the preferred learning style(s). Over the years different learning 'habits' that help benefit more from some experiences than from others. Since, this questionnaire will help pinpoint the learning preferences. There is no time limit to this questionnaire. It will probably take 10-15 minutes. The accuracy of the results depends on honesty. There are no right or wrong answers. If the residents agree more than disagree with a statement, they should put a tick. This questionnaire includes the scoring to be (activist-reflector-theorist-and pragmatist) and style preference (very strong-strong-moderate-low and very low). Activist learns well in new environments with activities and variety. Reflector learns well when opportunity to reflection and thinking is provided to them. Pragmatists learn well when they can apply their knowledge in practical life. Theorist learns well when they are able to generate and create ideas [6].

**Data analysis:** data were entered into a personal computer and were analyzed using STATA. Tests of significance (i.e. Fischer exact test) were applied. P-values less than 0.05 were considered as statistically significant.

**Ethical considerations:** this research project is approved from Institutional Review Board (IRB) in Primary Health Care Corporation, Qatar.

## Results

**Figure 1:**
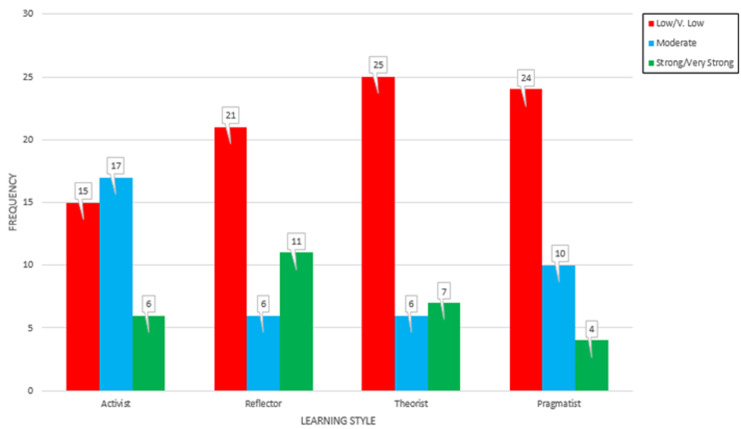
learning style pattern of residents

**Figure 2:**
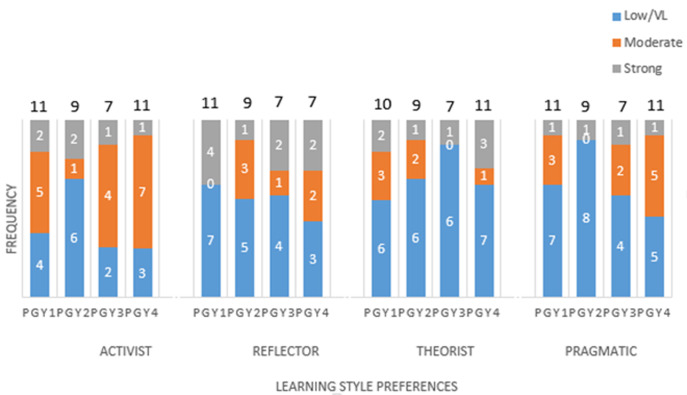
learning style preferences according to PGY level

Table 1 shows resident characters revealing 63.16% above age of 30 years, 68.42% were male, 68.42 were married, their distribution according to PGY level were 28.95% in PGY1, 23.68% in PGY2, 18.42% in PGY3, and 28.95% in PGY4. Figure 1 shows the learning style pattern of all PGY groups revealing that they are not follow specific pattern but rather mixed pattern. But generally, they tend to be in the following order: activist 60.5%, then reflector 44.7%, followed by pragmatism 34.2% and finally theorist 36.8%. Figure 2 shows relation between learning style and PGY level revealing that the predominant pattern in PGY1 is activist in 65% and theorist in 55% while PGY2 tends to be reflector in 45% and theorist in 35% and in PGY3 - 4 changed to be 70-75% activist and 40-55% (reflector and pragmatic). Table 2 shows no significant relation between resident character in the form of age, gender, marital status and PGY level on learning style.

**Table 1:** family medicine residents' characters

	Freq.	Percent
**Age**		
less than 30	14	36.84
More than 30	24	63.16
**Gender**		
Male	26	68.42
Female	12	31.58
**Marital status**		
Single	26	68.42
Married	12	31.58
**Residents level**		
PGY1	11	28.95
PGY2	9	23.68
PGY3	7	18.42
PGY4	11	28.95
Total	38	100

**Table 2:** relation between resident character and learning style

Character	Activist Low/VL	Mod/Str	Reflector Low/VL	Mod/Str	Theorist Low/VL	Mod/Str	Pragmatist Low/VL	Mod/Str
**Age**								
≥ 30 years	4	10	7	7	10	7	7	7
< 30 years	11	13	14	10	15	16	17	7
P value	0.3293 (NS)		0.7342 (NS)		0.5558 (NS)		0.2979 (NS)	
**Gender**								
Female	10	16	15	11	17	9	16	10
Male	5	7	6	6	8	4	8	4
P value	1.000 (NS)		0.7342 (NS)		1.000 (NS)		1.000 (NS)	
**Marital status**								
Married	11	15	13	13	17	9	17	9
Not married	4	8	8	4	8	4	8	4
P value	0.7281 (NS)		0.4862 (NS)		1.000 (NS)		1.000 (NS)	
**PGY Level**								
PGY1/PGY2	10	10	12	8	12	8	15	5
PGY3/PGY4	5	13	9	9	13	5	9	9
P value	0.1983 (NS)		0.7446 (NS)		0.5064 (NS)		0.1788 (NS)	

Low/VL = low to very low; mod/Str = moderate to strong; (NS) = not significant

## Discussion

This study was intended to identify the dominant style of a group of family medicine residents to explore the pattern of learning style and its relation with certain residents´ characters. In this study, residents tend to be in the following order regard learning style pattern: activist 60.5%, then reflector 44.7%, followed by pragmatism 36.7% and finally theorist 34.2%. Learning style combination of consultant was reflector theorist (56.7%), reflector pragmatist (16.7%), activist pragmatist (10%) and activist reflector (13.3%) while learning style combination of residents was activist theorist and activist reflector 22.5% each, reflector theorist 27.5% and reflector pragmatist 12.5% (p=0.023) concluding that consultants had a much better learning style and better time management strategies for improved learning [7]. We think the pattern of learning style would be different among residents with no specific pattern for certain residents so we can accept this difference.

This study shows that the predominant pattern in PGY1 is activist in 65% and theorist in 55% while PGY2 tends to be reflector in 45% and theorist in 35% and in PGY3-4 changed to be 70-75% activist and 40-55% (reflector and pragmatic). This in concordance of the study using the Honey and Mumford Learning Styles Questionnaire, in 2012 showed that the learning styles of male Saudi medical students shifted towards a predominantly “pragmatist” style over the first five years of undergraduate study [8]. This result matched our results which revealed changing of learning style from early residency (activist and reflectors) to late residency (activist and pragmatic also reflectors). This study concludes that no significant relation between resident character in the form of age, gender, marital status and PGY level on learning style. There was no association between genders and learning styles by study done in Saudi Arabia among dentist students [9]. It was also noted that some other studies, such as that of Shaw and Marlow, have observed no significant difference in Learning Styles between genders [10]. There was no significant association between age, gender or medical education status, and learning styles was found in a study done for internal medicine residency program [2]. This also match our results and may make us for searching other factors that may affect learning style such as original language, culture basis, location of undergraduate study, and academic background.

**Limitations:** this study has several limitations. First, the data were based on self-reports from residents and might be subject to recall errors. Second, the lack of demographic information about residents precluded investigation of the possible influence of residents´ characteristics (e.g., original language, culture basis, location of undergraduate study, and academic background). Third, the number of residents may be low for generalization of results; however, it may benefit local medical education authorities. Fourth, use of 80 item style questionnaire was too exhaustive and tricky for participants to understand and fill properly.

## Conclusion

Learning style assessment is important and can be used to determine which teaching modalities will be best accepted and most effective for family medicine residents which should be considered while planning, designing, and implementing their educational program.

### What is known about this topic

- Learning style is well studied in undergraduate students and different methods are being used to assess learning style.

### What this study adds

- This study adds to the literature about learning style among family medicine residents.

## References

[1] Dunn R, Giannitti MC, Murray JB, Rossi I, Geisert G, Quinn P (1990). Grouping students for instruction: effects of learning style on achievement and attitudes. J Soc Psychol.

[2] Adesunloye BA, Aladesanmi O, Henriques-Forsythe M, Ivonye C (2008). The preferred learning style among residents and faculty members of an Internal Medicine Residency Program. J Natl Med Assoc.

[3] Cook DA (2007). Web-based learning: Pros, cons, and controversies. Clin Med (Lond).

[4] Whitman N (1996). Learning and teaching styles: implications for teachers of family medicine. Fam Med.

[5] Sy A, Wong E, Boisvert L (2014). Learning behaviour and preferences of family medicine residents under a flexible academic curriculum. Can Fam Physician.

[6] Honey P, Mumford A (2000). The learning styles helper's guide.

[7] Mansoor S, Yousaf O, Rahman S (2019). Comparison of learning styles among post graduate residents and full time specialty clinicians pursuing higher educational degree. Pakistan Armed Forces Medical Journal.

[8] Guraya SS, Guraya SY, Habib FA, Khoshhal KI (2014). Learning styles of medical students at Taibah University: trends and implications. J Res Med Sci.

[9] ALQahtani DA, Al-Gahtani SM (2014). Assessing learning styles of Saudi dental students using Kolb's learning style inventory. Journal of Dental Education June.

[10] Shaw G, Marlow N (1999). The role of student learning styles, gender, attitudes and perceptions on information and communication technology assisted learning. Comput Educ.

